# The missing construct: Impathy

**DOI:** 10.3389/fpsyg.2022.726029

**Published:** 2022-10-06

**Authors:** Stefanie Neubrand, Jens Gaab

**Affiliations:** ^1^Division for Clinical Psychology and Psychotherapy, Faculty for Psychology, University of Basel, Basel, Switzerland; ^2^sysTelios Clinic, Wald-Michelbach, Germany

**Keywords:** impathy, impathic response, operational definition, Impathy Inventory, empathy

## Abstract

This article is about impathy (introversive empathy), understood as the ability to share in and understand one’s own feelings, which is considered a critical psychological construct relevant for the recovery and maintenance of mental health. However, while the ability to empathize with oneself has received considerable attention from the clinical community, this has not been paralleled by the same scientific scrutiny, which was subject to the ability to empathize with others. Impathy has not yet been operationally defined and thus has remained relatively unexplored, both conceptually and empirically. This work describes an operational definition of impathy with four dimensions: Perceiving, Meta-Position, Accepting Attitude, and Understanding. Issues of differentiation from related constructs are discussed and avenues of clinical applicability are explored, suggesting that impathy exists as a distinct human capacity, which can be assessed and which has important clinical implications. The paper closes with future directions, including the assessment of impathy and possible research questions.

## Introduction

The ability to perceive and understand one’s own feelings has been identified as a key component of mental health (Salovey and Mayer, 1990; Schutte et al., 2007). Fostering this ability, i.e., enabling people to empathize with their own experience, has been emphasized as a central goal in psychotherapy (e.g., Rogers, 1975; Kohut, 1984/2013; Bohart, 1991; Jordan, 1991, 2010; Barrett-Lennard, 1997; Neubrand, 2013; Watson et al., 2014; Riess, 2017), in particular as a method to overcome trauma (Barth, 1988; Greenberg et al., 1996; Moor, 2007; Sherman, 2014; Neubrand and Dietrich, 2017; Kress et al., 2018) and to promote personal growth (Rogers, 1975; Bohart, 1991). Interestingly, this clinical and epistemological interest has not yet found entry into psychological research. This may be due to the fact that no significant starting point has been established for conducting traditional psychological research on introversive empathy, called *impathy*.

Tracing the history of impathy, the German word “Einfühlung” was translated into English as “empathy” more than a century ago (feeling into; Titchener, 1909, quoted after Wispé, 1986, p. 315). Back then, this ability *to feel oneself into* something or someone encompassed a broader understanding of empathy than it does today, including its introversive side. Stein (1917), for example, postulated that empathy with one’s past, future, or fantasized “I” can be considered an introversive analog to empathy with others. Today, however, empathy is studied in traditional psychological science almost exclusively in terms of how people can share in and understand the emotional states of *others* (for a similar definition, see Decety and Moriguchi, 2007; for a review, see Cuff et al., 2016; Hall and Schwartz, 2019; Eklund and Meranius, 2021). This contrasts with contemporary psychotherapy which increasingly recognizes impathy as an approach of clinical relevance and applicability. Various directions in psychological treatment are discovering this introversive side of empathy to be a human capacity that enables people to relate adaptively to their own experiences rather than, for example, avoiding their own feelings and thoughts or criticizing and devaluing themselves. This advancement in psychological practice is based on the assumption that by developing impathy, people improve their well-being by empowering themselves to build a self-efficacious and growth-promoting relationship with themselves and their experiences.

## A history with many names

An online search was conducted to determine if there is existing literature on introversive empathy. This process was not intended to be an exhaustive review, especially since a systematic investigation of the literature on impathy has not been readily feasible. Impathy has rarely been the focus of published research; rather, observations about the importance of the development of impathy for the recovery and maintenance of mental health have stimulated attempts to describe this human capacity and led to the integration of impathy into work that had a different primary research goal. Therefore, we drew on our expertise to locate key scholars and literature sources in the field to gain initial insights into the nature of impathy.

Given this background, it is not surprising that introversive empathy has been referred to using various terminologies. Without a shared language for related phenomena, ambiguity arises, and advances in conceptualization and empirical research are hindered (Suddaby, 2010). For example, Snyder (1994) describes the human phenomenon of introversive empathy using the metaphor of an internal empathizer. Similarly, Schafer (1964) adopts the term intrapsychic empathy, while Jordan (1991) describes a self-empathy, and Kohut (1987) an attitude of expanded self-empathy. Furthermore, this concept has been described in many similar but different ways: By Snyder (1994) as “the attitude of compassion and curiosity regarding one’s own experience that enables one to be simultaneously conscious of feelings and detached from them” (p. 97) and by Schafer (1964) as “a readiness to recognize, a capacity to discern one’s own feeling states sensitively and to care about them; it is an aspect of benevolent or loving superego function as well as attentive ego function” (p. 294). By Jordan (2010) as “the ability to bring an empathic attitude to bear on one’s own experience,” by Kohut (1984/2013) as “the indirectly perceived experiences of one’s inner life” (p. 220), by Håkansson (2003) as “empathizing with […] (4) one’s own experiences in the past, or (5) one’s own experiences in the future” (pp. 44–45), and Barrett-Lennard (1997) proposed that it “involves a form of empathy turned inward, as the articulate ‘I-self’ devotes special listening attention to the wider underlying ‘organic’ self” (pp. 108–109), indicating a sketchy and partially tautological theoretical basis for understanding the psychological processing of introversive empathy. As a consequence, these attempts did not set out to contribute to the clarity of the construct and, in turn, did not lead to further development and clarification with regard to proximal concepts. However, several assumptions about the nature of impathy can be extracted from the definitions cited. The most basic postulate of these researchers appears to be the most salient, namely, the assumption that it is inherent in being human to have the capacity to relate to one’s own experiences empathically. James (1890/2014) made an important differentiation that has received renewed attention in psychological research because it can provide greater clarity in the dissonant discourse about “the self” (Swann and Bosson, 2010; Wozniak, 2018). James (1890/2014) argued that the self is composed of two main parts: “I” (self-as-subject) and “Me” (self-as-object). This understanding suggests that the “I” can relate to the “me” in a certain way. The ability to develop an “I”-perspective is a distinctive feature of being human. It allows the individual to understand themselves as part of an inner plurality as well as to act in it as a phenomenal subject “I” (Metzinger, 2003). This intrapsychic process opens up an inner space that is necessary to be able to engage with one’s own perceptual states, such as thoughts, feelings, and bodily sensations. Applied to the subject of the present work, it can be argued that by establishing a first-person perspective “I,” individuals are at the same time creating internal objects, e.g., a feeling of joy, to which they can direct their attention in a self-effective and autonomous way (Metzinger, 2003). In this way, a person becomes a phenomenal subject who can *feel themselves into their own experiences*, that is, the person becomes an active agent able to perform impathy actions. Accordingly, the ability to establish a first-person perspective “I” is considered a prerequisite for having an impathic experience (for similar discussion of introspection, see Metzinger, 2003).

Moreover, several of the definitions cited suggest that impathy and empathy share similar psychological processing patterns. Over the past two decades, the study of empathy has attracted considerable attention in the social neurosciences. This has led to new discoveries, both for conceptual approaches to empathy and for elucidating its neural features (Lamm and Majdandžić, 2015). An early and important finding of this research shows that the neural processing of perception of oneself versus perception of others share some common and some independent constituents (Decety and Jackson, 2004), suggesting that shared neural representations are central to how individuals understand foreign consciousness through themselves (Lombardo et al., 2010). For example, Cheng et al. (2010) conducted an fMRi-study examining the impact of taking “I”-related or other-related perspectives. They found that in both study conditions, that is, imagining oneself in a painful situation and imagining a loved one in a painful situation, elicited an enhanced neural response in networks associated with empathy. Moreover, embodied models suggest “that others’ emotional states are processed by re-enacting a representation of the same state in the observer.” (Dirupo et al., 2020, p. 16). This, in turn, implies that the degree of accuracy of the understanding of another’s state depends on the accuracy of the perception of one’s own affective states (Dirupo et al., 2020). Furthermore, the psychological literature on emotion experience emphasizes that the ability to empathize is facilitated when a person has had similar experiences in the past (Lambie and Marcel, 2002). Memory processing, again, is assumed to be linked to self-projection. Neuroscience research on self-projection speculates that imagining the future, remembering the past, and conceiving the view of another may all rely on operations of the same neural network (Buckner and Carroll, 2007). Although past experiences and imagination can facilitate empathy, this does not mean that they are necessary for an empathic experience (Gaesser, 2013). While our understanding of the neural basis of empathy has increased considerably over the past two and a half decades, deeper insight into the various psychological processes that enable humans to engage in this capacity remains an important research task in the field of social neuroscience. Still, regardless of whether the scientific inquiry addresses empathy or impathy, the human being itself remains the experiencing subject out of whose perceptual reality foreign as well as one’s own consciousness can be felt into, which inherently suggests similar ways of psychological processing of empathy and impathy.

Moreover, there are psychological researchers who claim that the capacity for impathy is an essential factor in the capacity to empathize with another (Barrett-Lennard, 1997; Håkansson, 2003). Again, these assumptions are rooted in the theoretical origins of empathy, because “only he who experiences himself as a person, as a meaningful whole, can understand other persons” (Stein, 1989, p. 116).

## Therapeutic considerations

It has been reasoned that a central function of the therapist’s empathy, in addition to establishing and maintaining a viable therapeutic relationship (Lambert and Barley, 2001), is to help clients internalize this way of relating to themselves (e.g., Jordan, 2010; Watson et al., 2014). Put differently: By modeling empathy with aspects and experiences of the client, the therapist helps the client learn how to relate to those aspects and experiences impathically. Being able to experience introversive empathy with aspects of oneself can lead to sustainable intrapsychic structural transformations, which, in turn, promote psychological well-being (Jordan, 1991). However, if it is not possible for a person to be impathic in a particular life situation, they may suffer some form of internal destabilization and dissociation (Bohart, 1991). Consequently, various clinical scientists see introversive empathy as an important component of psychological functioning (e.g., Rogers, 1975; Kohut, 1984/2013; Bohart, 1991; Jordan, 1991, 2010; Barrett-Lennard, 1997; Gilbert and Procter, 2006; Neubrand, 2013, 2014; Sherman, 2014; Watson et al., 2014; Neubrand and Dietrich, 2017; Riess, 2017). Furthermore, the development of impathy has been proposed to play a significant role in the treatment of a range of clinical issues, including eating disorders (Barth, 1988), dissociative identity disorder (Neubrand and Dietrich, 2017), moral injury in war (Sherman, 2014), self-criticism and forgiving (Gilbert and Woodyatt, 2017), self-injury (Trepal, 2010), and trauma (Banks, 2006; Moor, 2007; Kress et al., 2018).

In sum, these positions postulate that psychological health is a function of the ability to impathize with aspects of oneself, and that a significant lack of impathy can lead to both increases in and prolonged periods of dysfunctional arousal, thereby increasing vulnerability to psychological suffering. The development of impathy is expected to increase subjective well-being and health-promoting behaviors, and consequently, result in positive psychotherapy outcomes.

For example, relational-cultural therapy (Jordan et al., 1991)—a feminist therapeutic approach rooted in the psychodynamic tradition—is based on the idea that social connectedness contributes to the generation of a healthy “felt sense of self” (Jordan, 1997, p. 15), and that “self-empathy” is a crucial skill that needs to be strengthened for this (Jordan, 1991). In addition to counseling, for example, in the context of families and schools, relational cultural treatment models are used in the treatment of a wide range of mental health issues (Jordan, 2010). In a study with women diagnosed with eating disorders, short-term group relational therapy demonstrated as significant a reduction in bulimic and depressive symptoms as short-term group cognitive-behavioral therapy (Tantillo and Sanftner, 2003). From the perspective of scholars from the client-centered approach, an essential salutary function of empathy is to provide clients with a positive experience of how to deal with themselves in a way that enables them to navigate their lives with a sense of self-efficacy (Bohart, 1991). Consequently, Rogers (1975) concluded that the experience of being understood empowers the client to relate to themselves with increased empathy, thereby becoming “a more effective growth enhancer, a more effective therapist for himself” (p. 9). Likewise, empathy is considered fundamental to the client’s progress in emotion-focused therapy, especially because it promotes the client’s impathy (Watson, 2007).

These therapeutic perspectives share the common feature that they point to a process in which experiencing an empathic therapeutic environment facilitates the development of impathy within the client. In this understanding, change in psychotherapy goes hand in hand with how a client shapes their relationship with themselves. Watson et al. (2014) conducted a study in which depressed clients attended a weekly session of cognitive behavioral therapy or emotion-focused therapy for 16 weeks. These clients were asked to rate the empathy of their therapist. Results indicate that the perceived empathy of the therapist is associated with significant reductions in dysfunctional intrapersonal relating, e.g., decreases in self-criticism and self-blame.

In addition, Kohut (1987) emphasized the importance of teaching clients a health-promoting attitude toward themselves, so that they can develop a broader understanding of themselves: “This is essentially an attitude of expanded self-empathy – an expanded capacity for empathy with one’s own past and with aspects of oneself that one does not or not fully possess, including aspects of oneself that have not yet been expanded – in other words, with one’s own future possibilities” (p. 188). Another more recent therapeutic approach that identified the importance of increasing impathy is compassion-focused therapy (CFT, Gilbert, 2009). This approach postulates impathy as a crucial competency for the development of compassion. It combines training in impathy and empathy with, e.g., training in caring for well-being, and stress tolerance. A growing body of research points to the effectiveness of CFT across a wide range of well-being and mental health outcomes (for reviews see, Leaviss and Uttley, 2015; Craig et al., 2020).

Although there is an evolving recognition of impathy in the clinical community, interest in this human capacity has grown without accurately specifying the observed phenomenon into a definition that captures the underlying qualities and characteristics which would allow for careful evaluation. As a result, these assumptions have so far remained without thorough investigation and consequently without empirical significance. To address these limitations, the overarching aim of this article is to provide an operational definition of impathy that specifies its dimensional model to help clarify the construct and enable measurement and empirical research.

## From empathy to impathy

The operational definition of impathy presented here is the result of an initial validation process that aims at providing a conceptualization and operationalization of the new psychological construct impathy utilizing a theory-and empirically-based approach to facilitate traditional psychological research (Neubrand, 2021). This approach reflects the significance of accurate conceptualization as well as the fact that conceptualization and operationalization constitute different phases within a temporal cycle. Hence, the conceptual framework of a construct should be established before any measurement instrument is constructed (Zhang et al., 2016).

In simplified terms, impathy can be considered as an inwardly directed empathy. Thus, a basic premise of this work is to meaningfully apply insights from empathy research to the intrasubjective level of impathy. Although much of the literature on empathy highlights definitional differences and lack of construct clarity, hindering the progress of research on this psychological capacity, recent analyses suggest a growing consensus on critical themes in this field (Eklund and Meranius, 2021). One indication of the increasing conceptual consolidation in this field is the fact that highly relevant researchers are moving toward complex operational definitions that integrate these themes within a framework of interacting components (Hall and Schwartz, 2019). Thus, the aim of this article is not to review the myriad findings of empathy research or to define what empathy “is” or” is not.” Rather, the aim is to describe central themes of empathy research that guide the operational definition of impathy presented in this article.

Findings from this research describe empathy as a multifaceted construct that involves both affective (sharing in the experiences of others) and cognitive (understanding the experiences of others) components (Davis, 1983; Eisenberg et al., (1994); Decety and Jackson, 2004; Singer and Lamm, 2009; Zaki and Ochsner, 2012). Likewise, Decety and Moriguchi, 2007 define empathy as “the capacity to share and understand emotional states of others in reference to oneself” (p. 1).

Until recently, findings in social neuroscience implied that sharing experiences and understanding experiences represent two modes of empathy—often referred to as affective empathy or cognitive empathy—to achieve the same outcome, and that these two main sub-components represent neurally separate systems. However, the underlying research is impaired by difficulties, both in research methodology and conceptualization, which may complicate its valuable contributions to the extensive body of literature on empathy in psychology (Zaki and Ochsner, 2012). Moreover, study results show that these affective and cognitive components of empathy are neuronally dissociable only in relation to physical pain (Lamm and Majdandžić, 2015), but are interconnected when experiences are shared and mentalized in the context of more complex social tasks (Zaki and Ochsner, 2012). This illustrates the dynamically interrelated nature of the psychological processes underlying the experience of empathy in near-life social reasoning tasks (Zaki and Ochsner, 2012). Against this background, Zaki and Ochsner (2012) suggest “scientists to begin moving past ‘either/or’ conceptualizations of empathy’s processes as distinct, and toward a ‘when/how’ model, which posits that perceivers flexibly deploy multiple, interactive processes when they are relevant to current social goals and cues.” (p. 678).

For empathy to arise, it is necessary to focus enough attention on another person’s condition so that the perception of that condition becomes possible (Preston and de Waal, 2002). To perceive another’s state is not to merge with the other’s feelings, but to enter into the inner world of the other “as if” it were one’s own and to meet them with acceptance and openness (Rogers, 1959). That is, empathy involves the intention to direct one’s attention to another’s experience in a particular way (Zahavi, 2008) while maintaining sufficient awareness that the source of the shared experience originates in the other and not in oneself (e.g., Decety and Jackson, 2004). For example: “I share in your sadness and am aware that the source of sadness is within you.” In fact, drawing on Carl Rogers’ “as if” stance in empathy, many researchers (e.g., Decety and Jackson, 2004; Decety and Lamm, 2006; de Vignemont and Singer, 2006; Cuff et al., 2016) hold that the ability to self-other discrimination is a critical criterion for empathy.

The interaction between first-person experience and third-person experience through affective sharing enables a person to understand consciousness outside of oneself (e.g., Stein, 1917; Preston and de Waal, 2002). For example, Decety and Moriguchi (2007) suggest that empathy encompasses “some minimal recognition and understanding of another’s emotional state.” (p. 1). According to Leiberg and Anders (2006) “empathy is the ability to perceive and understand other people’s emotions and to react appropriately.” (p. 419). To ensure that sharing in another’s state does not lead to personal distress and self-focused reaction, empathy encompasses (meta)cognitive mechanisms to regulate one’s emotions (Hoffman, 1982; Eisenberg et al., 1994; Decety and Jackson, 2004). Accordingly, various empathy researchers suggest that empathy is a process in which affect and cognition are mutually interrelated (Cuff et al., 2016). Empathy is seen as a crucial part of a process that can lead to an empathic response. When empathizing with a person who is suffering, such an empathic response can elicit compassion for another (Singer and Klimecki, 2014). Empathy and compassion are identified as essential for the development of morality and helping behavior (Eisenberg and Miller, 1987; Batson and Shaw, 1991; Hoffman, 2008; Goetz et al., 2010; Masten et al., 2011). The extent to which a person is capable of being empathic varies according to situational and personality factors (Akitsuki and Decety, 2009; Gonzalez-Liencres et al., 2013) as well as on the perceived social relationships among people (Fan and Han, 2008).

Similarly, for impathy to arise, sufficient attention must be directed to one’s own state so that the perception of that state becomes possible. To regulate distress and navigate between internal states, the impathic process is understood to require metacognitive skills. In this way, both a merging with and a disconnection from one’s experience can be prevented. If there is a significant loss of internal contrast between the phenomenal subject “I” and certain states, individual aspects of the experience (e.g., shame) move to the fore, and the likelihood of overidentification with these inner states and the perpetuation or intensification of personal distress grows. To develop an accurate picture of inner phenomena, the impathic process involves the ability to experience with openness and acceptance. In the absence of this attitude, individuals may seek to adjust their subjective experiences to match their ideal of themselves, which in turn may result in maintaining or increasing stressful emotions due to self-criticism (Blatt et al., 1976). Developing a deeper understanding of one’s experience may be part of the impathic process. With the awareness that the source of one’s own experience represents specific experiences rather than the person as a whole, a direct transfer of understanding takes place in the process of impathizing.

Accordingly, impathy is defined as a process of active intrapsychic engagement that involves the ability to perceive and understand one’s own internal states and circumstances with an accepting attitude, while being sufficiently aware of the fact that the source of the perceived experience represents individual feelings, thoughts, and sensations rather than the person in their complex entirety. Impathy is part of an intrapersonal process that can elicit an impathic response, e.g., self-compassion and introversive helping behavior in times of suffering. This definition of impathy reflects the multidimensional nature of empathy and explicitly refers to the significance of subjectivity in impathic experience which is embedded in an internal and external context of meaning.

## Structure and process of impathy

It is unlikely that a single factor can be found to explain a human phenomenon of such complexity, thus the goal in operationalizing impathy is not to find just one, but several meaningful factors. Drawing on conceptualizations of empathy, the nature of impathy is best to be understood as multifaceted with interdependent processing of several dissociable dimensions and their underlying psychological processes. Four interdependent dimensions of impathy have been identified: Perceiving, Meta-Position, Accepting Attitude, and Understanding.

The first dimension involves the perception of one’s own physical and psychological phenomena, thereby turning the focus of attention inwards and engaging with one’s own states. The second dimension includes the ability to develop and maintain sufficient mental flexibility in relation to one’s inner experiences. The third dimension comprises a particular attitude in which attention is directed to one’s own experience, an attitude characterized by openness and acceptance. The fourth and final dimension refers to understanding and contextualizing one’s own sensations. This view implies that none of the four subdimensions is sufficient by itself to enable the human capacity for impathic processing. For example, in the absence of adequate metacognitive activity, perceiving an emotion (e.g., fear) may cause the individual to experience a very high level of arousal stimulated by their own affect, resulting in personal distress. The four subdimensions of impathy are specified in the following.

### Perceiving

To generate an impathic experience, a person directs their attention inward to their present sensations – temporarily perceiving and participating in their thoughts, feelings, physical sensations and their own circumstances.

Impathy can be initiated by a variety of situations. It can be activated more or less automatically, e.g., when I am injured in an accident or when a sad memory suddenly appears in my mind’s eye. It can also be elicited intentionally in response to a person purposely seeking to realize an impathic process. For example, when a person sits in front of their sad “I” in a therapeutic chair work and empathizes with it, or when a person has an imaginative encounter with themselves in a hypnotherapeutic session (Neubrand and Dietrich, 2017). Regardless of how the perception is triggered, in the course of the impathic process the person becomes an active agent engaging with a perceptible internal entity, such as a feeling of fear in the chest (for discussion of attentional agency, see Metzinger, 2003). Such conscious emotions as fear are real and are a form of perception. The perception of an emotion is analogous in nature to other perceptions. This means that experiencing an emotion, like other perceptual performances, involves “bottom-up” processes, but it is also the result of complex processing of raw material, underlying conceptual frameworks, learning experiences, and a person’s context (Russell, 2009). Through the perception of emotions, people receive information that enables them to understand their own mental states. Emotions are embodied and represent perceptible physical changes, i.e., to perceive ourselves we use our bodies (Prinz, 2004). Being in an emotional state means being in a certain phenomenological state (Lambie and Marcel, 2002). All of the phenomenal states that a person can perceive at a certain moment qualify as content for intrapersonal processing (Swann and Bosson, 2010) and thus as the subject of impathic processing. In order for a person to be able to process first-order phenomenology as an impathic agent, they should be able to focus a sufficient amount of attention on their own experiences. Ingram (1990) defines self-focused attention “as an awareness of self-referent, internally generated information” (p. 156) which includes phenomenal information, for example, about physical states, memories, and feelings.

In psychological practice, it is usually expected that people possess at least a minimum level of contact to their own feeling states. There are, however, people who find it very challenging to perceive and understand their own emotions which is considered a key characteristic of alexithymia. Alexithymia is associated with a broad spectrum of disorders that involve impairments in accessing and utilizing personal experiences as a reference for one’s behavior (Ogrodniczuk et al., 2011; Bird and Cook, 2013) and, in sum, presumably imply deficits in impathy. Alexithymia is a personality trait which should be conceptually linked to impathy though located at the opposite end of a shared continuum.

### Meta-position

Impathy also refers to the ability to perceive one’s own phenomena and at the same time not fuse with them—by regulating the inner movement between more proximal and more distal experiences. In this way, a person can experience their autonomy and flexibility in navigating an impathic encounter.

Skills in meta-level processing should provide the subjective experience of intentionally realizing an internal act as a phenomenal “I,” i.e., keeping the focus of attention on a self-chosen aspect of one’s own experiences for a specific time and in a specific way (Metzinger, 2003). The ability to develop a meta-position allows the person to create an internal “in-between” in order to relate to their own phenomena purposefully (Gonçalves and Ribeiro, 2012). Purposeful intrapersonal behavior here means that the impathic process is guided by an executive quality. A central aspect of executive functioning is to enable a person to choose *how* to deal with themself (Baumeister, 1998).

Consequently, one prediction of this model is that increases in impathy are associated with improvements in meta-level processing. Skills in meta-level processing provide greater psychological flexibility in dealing with experiences (Decety and Jackson, 2004). Metacognitive skills are considered to be of major importance for mental health (Bernstein et al., 2015) and change processes in psychotherapeutic treatment (Teasdale et al., 2002) because the ability to empirically distance oneself from oneself provides an internal context in which a person can develop healthier communication with themself (Cunha et al., 2011). Consequently, it is hypothesized that the development of impathy facilitates the development of more flexible forms of intrasubjective relating as the person learns to regulate their closeness and distance to their emotional states to allow for impathic experience.

### Accepting attitude

In impathic experience, the person engages in an active process to grasp their feelings in a certain way. This way of phenomenal processing involves allowing one’s own feelings, thoughts, bodily sensations, and situation to become the focus of one’s attention without evaluating them as to whether they are pleasant or unpleasant; in other words, “adjusting” them as little as possible to one’s ideal conception of oneself and of reality.

Hayes et al. (2006) define acceptance as actively attending to one’s own experience while avoiding any dysfunctional efforts to modify it. Acceptance characterizes active intrapersonal behavior, as the person intentionally attempts to engage in an open and non-judgmental contact with their own feelings and thoughts (*cf.* Bishop et al., 2004). Impathy can be understood as an intrapsychic process that is neutral toward the content of one’s experience but intentional toward the way that content is processed. The adoption of an accepting attitude in the development of impathy could lead to a reduction of inner criticism and judgment. Research suggests that self-criticism is associated with depression (Blatt et al., 1976; Blatt and Zuroff, 1992). Acceptance-based therapy approaches integrate these insights by educating and training people to perceive their thoughts and emotions without judging them or getting carried away by them (Hayes and Feldman, 2004). Accepting oneself is considered a key aspect of well-being (Ryff, 1995) and is usually accompanied by distancing oneself from one’s experience. However, while promoting internal distancing mechanisms can lead to greater acceptance and the other way around, one difference between these approaches is that distancing oneself from challenging personal events does not automatically translate into acceptance of those events (Herbert and Brandsma, 2015).

### Understanding

Impathy is about intentionally engaging in inner contact, thereby increasing the level of accuracy in the encounter with oneself—by allowing a particular inner phenomenon to become the focus of affective sharing.

The ability to share in one’s own inner experience (e.g., a feeling of anxiety, an imaginary success) should be necessary in order to develop a deeper understanding of one’s own experience. By focusing attention on a particular internal phenomenon (e.g., a tightness in the chest), this phenomenon takes on a figurative character in comparison to the surrounding inner perceptual context (Silvia and Gendolla, 2001), thus forming a contrast within the stream of consciousness and becoming an object available for internal processing (Metzinger, 2003). In this way, the accuracy of understanding of this phenomenon can be increased (Silvia and Gendolla, 2001). The contents of inner phenomena possess a functional property that can be empathized (for an example of memory, see Stein, 1917) as can the way in which a person relates to their experiences. This implies that, in addition to the understanding that is revealed in one’s own experiences, it is also possible for a person to gather meta-knowledge about *how* they process their own feelings, memories, longings, etc. (Metzinger, 2003).

Based on this conceptualization, it can be speculated that impathizing may enable people to sharpen their self-knowledge. Strengthening introversive empathy over time is likely to lead to a more realistic assessment of one’s own capabilities and limitations (Gilbert and Woodyatt, 2017), creating favorable conditions for coping with future challenges and effective problem solving. Social problem solving (McCabe et al., 1999) correlates with higher self-esteem as does greater and consistent self-knowledge (Campbell, 1990; Stinson et al., 2008). Interestingly, the associations between self-knowledge and self-esteem do not necessarily seem to be direct. One important moderating variable for the effects of self-knowledge on self-esteem is certain self-beliefs (e.g., “I am a good friend” or “I am a failure”). The influence of a person’s self-beliefs is, in turn, affected by their context (e.g., cultural) and their expectations of what their ideal self should be (Showers and Zeigler-Hill, 2006). Accordingly, self-esteem defines how a person judges their own worth as a person (e.g., MacDonald, 2012) and is based on the extent to which a person appreciates and accepts themselves (Rosenberg, 1965). Accepting oneself and one’s own experiences is, in turn, considered a crucial characteristic of impathy.

Against this background, it seems reasonable to hypothesize that the ability for impathy is positively associated with self-esteem. In addition, understanding one’s own emotional states increases one’s ability to empathize with others (Preston and de Waal, 2002). Congruently, researchers suggest that impairments in empathy are associated with alexithymia (Decety and Moriguchi, 2007; Ogrodniczuk et al., 2011; Bernhardt and Singer, 2012).

## From impathy to the impathic responding

In the course of an impathic experience, a person develops closeness with themself and gains access to a broader spectrum of their own reality. They discover aspects they were not aware of before and develop a richer understanding of themselves, which enables them to react more adequately to their personal phenomena and to utilize the impathic experience as a reference for their behavior.

An example of such an experience could be: “I now understand that I was very alone when I sat at my dying partner’s bedside.” This deeper understanding can be irritating at first, and it can be a catalyst for changing the way a person reacts to their experiences. It is the source for the change of a person’s self-concept (Rogers, 1975). This change, in turn, motivates a person to modify their behavior so that it is consistent with their evolving sense of self because, as Rogers further argues, people strive for a feeling of inner congruence. The impathic experience, therefore, should provide an internal reference to which a person can turn for guidance on how to respond skillfully to their inner conditions and circumstances (*cf.* Rogers, 1975; Bohart, 1991). One such response may be, “I feel compassion for my past ‘I,’ because now I understand that I, too, needed someone to be there for me.”

Accordingly, impathy is part of an intrapsychic process that can trigger an impathic response. This means that in this work, impathy is understood as a singular conceptualization which implies the separation of impathy and a response behavior. Since every human experience is embedded in a personal situation, impathy and the impathic response can be assumed to be related to the individual’s perception of their context and personality. Whether an impathic process and response are appropriate or inappropriate, moral or immoral, is subject to the individuality and autonomy of the impathic person.

In summary, impathy comprises four core dimensions: Perceiving, MetaPosition, Accepting Attitude, and Understanding. Impathic experience forms an internal reference that provides guidance in shaping one’s own behavior. When a person experiences suffering, impathy should imply a behavioral tendency toward self-compassion and introversive helping behavior.

## Similarities with and differences to related constructs

Based on the presented understanding of impathy, several other constructs show theoretical proximity as well as differences which shall be described in the following. First, impathy shows similarity to constructs encompassing *affective* experiencing. In this sense, impathy could be seen as a mediating factor for the emergence of self-compassion (feeling concern for oneself; Gerber and Anaki, 2021), as an accurate understanding of one’s own distress should facilitate the development of compassion for oneself. Compassion, in turn, is an important factor in eliciting helping behavior aimed at alleviating suffering (Goetz et al., 2010). Consequently, increasing impathy should be associated with an increase in introversive helping behavior, especially when mediated by self-compassion. According to Neff (2003b), self-compassion entails three components: self-kindness vs. self-judgment, common-humanity vs. isolation, and mindfulness vs. over-identification. A growing body of research shows associations between self-compassion and well-being (for a review, see Zessin et al., 2015) and indicators of mental health (for a review, see MacBeth and Gumley, 2012). However, although these constructs may be related, there are good reasons to distinguish between them. Impathy, building on insights from empathy research (e.g., Eisenberg and Miller, 1987; Bohart, 1991; Singer and Lamm, 2009; Decety and Michalska, 2010), is understood as a “feeling with oneself,” whereas self-compassion is rather a “feeling for oneself.” “Feeling with” indicates that the feelings one experiences are in some way congruent between the phenomenal “I” and the primordial, i.e., original, inner state (e.g., “I feel joyful when I share the joy of my past ‘I’”; *cf.* Stein, 1917). “Feeling for” oneself, on the other hand, indicates an incongruence between the feelings one has with respect to the phenomenal “I” and the primordial inner state (e.g., “I feel concern for myself now that I understand the sadness of my past ‘I’”). Accordingly, “self-compassion” should be located in a common field with impathic response. Impathy, however, is not exclusively concerned with the experience of suffering. Fan et al. (2011) identified a broad range of emotions that can trigger empathy, including anxiety, anger, happiness, pain, and sadness. It stands to reason that there will be different impathic responses depending on the affective state a person is impathizing with (e.g., self-compassion when grieving for a loved one, happiness when remembering a joyful moment).

While empathy is conceptualized as the sharing of affect, the emotion shared, although it may feel similar, is still different from the emotion evoked in the empathic observer (Singer, 2006). With impathy, the term sharing refers to a person sharing a part of their own experiences (e.g., fear), which implies that impathizing goes beyond affective experiencing and also shows associations with constructs involving *cognitive* capacities. That is, impathy, as conceptualized here, involves both an affective component, to establish an internal relationship through sharing, and a cognitive component, to distinguish between the phenomenal subject “I” and its discrete personal experiences. This metacognitive ability to regulate the interplay of proximity and distance to internal phenomena should be similar to constructs such as decentering (Safran and Segal, 1990), cognitive defusion (Hayes et al., 2012), or mindfulness (e.g., Bishop et al., 2004). They all describe metacognitive capacities that enable people to navigate their focus of attention in a specific way and to tolerate aversive personal phenomena (for a review of decentering-related constructs, see Bernstein et al., 2015). Moreover, this cognitive aspect distinguishes between empathy and emotional contagion (Decety and Jackson, 2004). If this metacognitive capacity is significantly lost, a person may become absorbed by their own states and instead of self-compassion the development of self-pity becomes likely (Neff, 2003a). People who feel pity for themselves are prone to overshare their own difficulties and become absorbed in their feelings and thoughts (Stöber, 2003).

Impathy may also be similar to psychological concepts that include self-reflective attention, such as introspection (looking inward with the goal of “examining the contents of one’s mind”; Wilson, 2002, p. 159), objective self-awareness (a person becomes the object of their reflection; Duval and Wicklund, 1972), private self-consciousness (“the consistent tendency of persons to direct attention inward”; Fenigstein et al., 1975, p. 522), and self-monitoring (“self-observation and self-control guided by situational cues to social appropriateness”; Snyder, 1974, p. 526). What separates impathy from these psychological constructs is that the latter are used to evaluate one’s mental and emotional content. However, as has been discussed, analyzing and judging should be in contrast to impathy. Their common feature is, therefore, likely to be an increase in understanding. For example, understanding feelings through empathizing differs substantially from understanding through mentalizing (Singer, 2006). Similarly, understanding one’s own feelings through affective sharing *via* impathy should be different from understanding through self-reflection, e.g., *via* introspection. That is, impathically understanding feelings of shame should be different from introspectively trying to understand what personal factors (e.g., past behaviors, character traits) have caused one to be in a shameful situation (e.g., “If I had not been lazy and prepared well instead, I would not have embarrassed myself in front of my colleagues”). Both mentalizing (Singer, 2006) and introspection lack affect and physicality. This may also be a key difference between impathy and the theory of self-awareness, as objective self-awareness involves reflection and cognitive analysis of the self (Wicklund, 1975). When an individual is objectively self-aware, they tend to evaluate themselves and to compare actual aspects with their ideal conceptions of themselves. Increased awareness of negative discrepancies is likely to trigger self-criticism as well as the avoidance of self-awareness (Wicklund, 1975; for a review, see Silvia and Duval, 2001). In contrast, again building on findings from empathy research, it is assumed that, in addition to cognitive aspects, the ability to resonate affectively with one’s own experiences without judgment is an important gateway to impathy.

In conclusion, it can be reasoned that there are functional differences between impathy and related constructs. Impathy, as defined here, includes both an affective component and a cognitive component. Although impathy may lead to emotional (e.g., self-compassion) and/or behavioral responses (e.g., introversive helping behavior), these implications are not part of impathy itself, but reflect possible outcomes of engaging in an intrasubjective process that begins with feeling oneself into one’s own experience.

## Implicit and explicit ways to promote impathy

The observation by various scholars that people are able to learn to empathize with themselves is highly relevant to psychological practice because it reveals a person’s potential to become an impathic agent in their own right. It is theorized that it is through one’s own affective sharing that the person is enabled to have certain possibilities, e.g., the intrapsychic possibility of (re)connecting with previously rejected or dissociated experiences by turning to them in an impathic process (see Bohart, 1991; Jordan, 1991; Neubrand and Dietrich, 2017 for the example of traumatic experience). Such impathic discoveries, it is further hypothesized, may hold the potential to change a person’s psychological structure (Jordan, 1991). As discussed earlier in this work, several researchers have suggested a process in which the experience of the therapist’s empathy implicitly influences the way clients relate to themselves. That is, they assume that the experience of an empathic context in therapy can give rise to something new within the client, something that the client is able to grasp and integrate into themselves by establishing an impathic context. This perspective offers a coherent explanation of how empathic characteristics of the therapeutic alliance influence a person’s mental content, pointing to an intersubjective process by which individuals integrate qualities of the other into their own concept of self (Aron et al., 1991).

Consistent with this, research shows that closeness in interpersonal relationships generates an expansion of oneself, in that one’s self-concept grows to include new attributes (Aron et al., 1995).

For example, many individuals struggling with bulimia display a very self-critical attitude and are “therefore unable to empathize with themselves” (Barth, 1988, p. 272). For the affected person, the therapist’s empathy often represents an opportunity to have a new interpersonal experience (Barth, 1988). Adverse self-evaluations are also a common consequence of rape. The therapist’s empathic statements act as a mirror reflecting empathy in contrast to the client’s self-critical statements. This empathic echo creates space for a different view of oneself, understanding that suffering has been inflicted on one (Moor, 2007). Self-judgment and self-destruction can then be let go of and “self-empathy and compassion are expected to follow, and to give way, in turn, to affirming views of self” (Moor, 2007, p. 26). According to Barth (1988), “such” self “empathy is necessary before the feelings can be integrated into the individual’s overall sense of self” (p. 272). In summary, the experience of second-person empathy is thought to *implicitly* facilitate the development of first-person empathy (*cf.* Sherman, 2014), namely impathy.

If, however, the ability to impathize is of such great importance for mental health and therapeutic change, the question arises as to how it can be *explicitly* addressed, i.e., whether there are ways to target the client’s impathy in psychotherapy that go beyond implicit learning experiences. For example, the two-chair intervention aims to help clients develop impathy and dissolve their self-critical beliefs (Barnard and Curry, 2011). Against this background, research on self-compassion suggests that this intervention, by aiming to promote impathy, is highly beneficial for increasing self-compassion (Neff et al., 2007). Consequently, Neff et al. (2007) conducted a study in which they used the two-chair technique and asked participants to recall a situation in which they had been critical of themselves, showing that enhanced self-compassion was correlated with enhanced well-being. These findings could be understood that impathy is a strong proximal determinant for the development of self-compassion. Moreover, as hypothesized for self-compassion (Luoma and Platt, 2015), impathy may be implicit to “self as context,” a key principle in Acceptance and Commitment Therapy (Hayes et al., 2006), because “self as context interventions often focus on increasing more flexible, empathic ways of relating to oneself” (Luoma and Platt, 2015, p. 99). In Buddhist traditions, empathy is considered a human capacity that can be cultivated explicitly in relation to oneself and in relation to others, e.g., through loving-kindness meditations (Kristeller and Johnson, 2005) which are increasingly incorporated in the treatment of mental health problems. Neubrand and Dietrich (2017) provide another example of the application of impathy in psychotherapy by integrating both indirect and direct ways to promote impathy in the treatment of people with dissociative identity disorder. In summary, the capacity for impathy enables a person to meaningfully engage in intrapersonal interactions with past, present, future, and imagined experiences, thereby enabling them to process both everyday and otherwise unbearable experiences to improve and maintain their mental health.

### Future directions

Whether the capacity for impathy shares similar processing patterns with empathy, or whether it can be enhanced by specific interventions, will be one of the tasks of future impathy research. Along with the development of rigorous conceptualization, appropriate measurement instruments are needed to enable traditional psychological research on impathy. Considering the assessment of related constructs, the measurement of impathy could follow well-established methods. For example, recent developments in social neuroscience have opened up new ways to study and quantify different aspects of empathy, such as empathy in relation to physical pain. Neuroimaging studies could be used to measure brain activity as a measure for studying impathy and its neural substrates. Another useful way to investigate the nature of impathy may be provided by electrophysiologic approaches. For example, researchers could investigate whether there are temporal dynamics in the psychological subprocesses of impathy. Recent research on empathy in pain, using electroencephalography to study event-related brain potentials, shows that neural activity linked to affective sharing arises at an earlier stage than cognitive understanding (Fan and Han, 2008). To further increase construct clarity, it would be of interest to assess relevant constructs such as self-compassion, objective self-awareness, self-esteem, mindfulness, and alexithymia in order to discriminate them from impathy and to explore possible associations.

Because the capacity for impathy is considered critical for mental health recovery and maintenance, it may be of particular interest to examine impathy in clinical settings. However, many clinical researchers lack access to advanced technology such as functional magnetic resonance imaging (fMRI) (Gerdes et al., 2011). Therefore, the use of self-report questionnaires, which can be easily and economically conducted, remains a very important approach for traditional psychological research and, consequently, for future research on impathy. Based on the operational definition of impathy presented in this paper, the authors constructed a self-report questionnaire, the Impathy Inventory (Neubrand, 2021). The Impathy.

Inventory measures a person’s capacity for impathy using 20 items distributed evenly among the proposed subcomponents of Perceiving, Meta-Position, Accepting Attitude, and Understanding. Preliminary validation studies suggest that the Impathy Inventory is an economical and psychometrically sound self-report instrument.

Substantial research will be needed to further evaluate the new psychological construct of impathy and to investigate its presumed pivotal role for mental health.

## Conclusion

As awareness of the clinical significance of impathy increases, so does the need for thorough investigation in this field. Assumptions about experiential manifestations and theoretical descriptions in the clinical literature provide initial clues about the nature of impathy. The task, therefore, is to facilitate basic scientific research so that understanding about this psychological construct can grow and, in turn, support psychological practice. To provide a solid foundation for empirical research, a conceptual basis of impathy is needed that will enable the construction of valid measurement instruments. This will allow for the examination of previous assumptions as well as emerging research questions about impathy, both in terms of its empirical properties and its potential significance for the advancement of psychotherapy. This work proposes a testable operational definition of impathy with four dimensions: Perceiving, Meta-Position, Accepting Attitude, and Understanding. Based on this conceptualization, the authors developed and initially evaluated a four-dimensional self-report instrument to measure interindividual differences in the ability to impathize, the Impathy Inventory (Neubrand, 2021). As such, together with the conceptual work presented here, the foundation has been laid for empirical research and clinical advancement on impathy.

## Data availability statement

The original contributions presented in the study are included in the article/Supplementary material, further inquiries can be directed to the corresponding author.

## Author contributions

SN had the original idea to define a concept to assess introversive empathy and then conducted several empirical studies as well as started to research the embedment of this concept in existing theories and approaches. JG supervised this work and served as a reflecting partner. SN wrote the draft of the manuscript, which was commented upon and revised by JG. All authors contributed to manuscript revision, read, and approved the submitted version.

## Conflict of interest

The authors declare that the research was conducted in the absence of any commercial or financial relationships that could be construed as a potential conflict of interest.

## Publisher’s note

All claims expressed in this article are solely those of the authors and do not necessarily represent those of their affiliated organizations, or those of the publisher, the editors and the reviewers. Any product that may be evaluated in this article, or claim that may be made by its manufacturer, is not guaranteed or endorsed by the publisher.
